# Determinants of COVID-19 Vaccine Acceptability among Healthcare Workers in Kenya—A Mixed Methods Analysis

**DOI:** 10.3390/vaccines11081290

**Published:** 2023-07-27

**Authors:** Victor Mudhune, Ken Ondeng’e, Fanuel Otieno, Derick B. Otieno, Collins M. Bulinda, Ikechukwu Okpe, Sarah Nabia, Naor Bar-Zeev, Omondi Otieno, Chizoba Wonodi

**Affiliations:** 1 Kenya Medical Research Institute, Centre for Global Health Research, Kisumu P.O. Box 1578-40100, Kenya; kondenge@gmail.com; 2 Capacities for Health and Social Policy, Nairobi P.O. Box 32175-00800, Kenya; fanuel@chasp.co.ke (F.O.); derick@chasp.co.ke (D.B.O.); bulinda@chasp.co.ke (C.M.B.); omondi@chasp.co.ke (O.O.); 3 Direct Consulting and Logistics, Abuja 904101, Nigeria; okpe.ikechukwu@dclnigeria.com; 4 International Vaccine Access Center, Johns Hopkins University, Baltimore, MD 21231, USA; snabia1@jhu.edu (S.N.); cwonodi1@jhu.edu (C.W.)

**Keywords:** COVID-19, vaccination, vaccine acceptance, 3C model, healthcare workers, Kenya

## Abstract

Healthcare workers (HCWs) were a priority group for COVID-19 vaccination. Adopting the World Health Organization’s 3C and the expanded 5C vaccine hesitancy models, we assessed the factors associated with COVID-19 vaccine acceptability among HCWs in Kenya. In a mixed methods study, respondents were from eight selected counties across the country. An online survey (n = 746), key informant interviews (n = 18) and focus group discussions (n = 3) were conducted. The data were analyzed concurrently. Quantitative data showed that all the 3C antecedents were strong predictors of vaccine acceptability. The association of vaccine acceptability was strongest with convenience (aOR 20.13, 95% CI 9.01–44.96), then complacency (aOR 10.15, 95% CI 4.63–22.21) and confidence (aOR 6.37, 95% CI 2.90–14.02). Marital status was a significant independent factor associated with vaccine acceptability (aOR 2.70, 95% CI 1.20–6.08). Qualitatively, convenience presented as the no-cost availability of vaccines at the health facilities, whereas non-complacency manifested from the first-hand observed experience of COVID cases, and the need to protect oneself and family members. Confidence was mainly attributed to increased knowledge, resulting from multiple training sessions and trust in regulatory authorities. Other social factors including workplace pressure, religion and misinformation had a role in influencing HCW vaccination decisions. In the background of a pandemic, the 3C model is a strong predictor of vaccine acceptability, and making the vaccines easily available and convenient to HCWs significantly impacts their uptake.

## 1. Introduction

The COVID-19 outbreak was declared a global pandemic by the World Health Organization (WHO) in March of 2020 [1]. Since then, the pandemic has spread across the globe, with about 650 million confirmed cases and 6.6 million deaths as of 23 December 2022 [2]. Given the scale of COVID-19 infections, SARS-CoV-2 variants have emerged [3], leading to variants of concern, largely characterized by increased potential of faster spread, severe health outcomes, escaping current diagnostic tests or resistance against available vaccines [4]. The persisting threats of emerging variants of concerns leading to subsequent outbreaks [3,5] underlines the importance of an effective vaccine coupled with high vaccine acceptability to control the infection.

Healthcare workers (HCWs) accounted for a significant number of initial COVID-19 cases [6], and are both potential victims and propagators of the infection. Protecting HCWs from SARS-CoV-2 infection is beneficial not only to themselves, but also their social contacts and the healthcare system. WHO prioritized HCWs for COVID-19 vaccination [7], acknowledging that addressing HCWs’ vaccine hesitancy and improving vaccine acceptability is a crucial ingredient in addressing vaccine hesitancy within their local community. Vaccine hesitancy among HCWs may result in a variety of negative effects, including affecting the behavior of the general population and challenges with maintaining human resources for health needed to fight the pandemic [8]. 

Kenya commenced its COVID-19 vaccination program in March 2021, mainly focusing on front line workers including HCWs. In this phased rollout of COVID-19 vaccination, HCWs were deemed a significant target group due to workplace exposure and the need to maintain the human resources for health against the pandemic. However, vaccine hesitancy remains a significant challenge in light of conspiracy theories [9]. WHO qualifies vaccine hesitancy as “complex and context specific, varying across time, place and vaccine type”, and is largely influenced by psychological determinants such as complacency (risk perception), convenience (level of constraints) and confidence [10]. COVID-19 vaccine acceptance has been shown to be lower among HCWs in low- and middle-income countries compared to those from high-income countries [11,12]. Studies have shown varying rates of acceptance among HCWs globally, with COVID-19 vaccination hesitancy ranging from 4.3 to 72% [11,13], while vaccine acceptability has been shown to range from 27.7% to 78.1% [12]. In Kenya, vaccination intention was associated with the belief in COVID-19 vaccine safety and risk management by the government [14], and vaccine hesitancy was less among physicians, and more likely among those with less than the median income, younger age, and those with safety and risk concerns [15].

Vaccinating a critical mass remains the greatest hope in fighting the COVID-19 pandemic, and future emerging variants [5]. Reaching the critical mass largely depends on addressing vaccine hesitancy at the community level, and building health systems that support vaccination program implementation [16]. HCWs are critical in supporting both initiatives; hence, continued assessment of vaccine acceptability and attitudes among HCWs is important in addressing the root causes of vaccine hesitancy and improving population uptake [11]. Pre-vaccine introduction evaluations of COVID-19 vaccine acceptance were largely based on self-reported intention to vaccinate, which has been shown to differ from actual uptake [17]. Post-vaccine deployment studies provide an accurate measure of acceptability, and subsequently, country-level analyses are needed to select the appropriate intervention to promote vaccine uptake. Individual’s psychological factors preceding vaccination behavior are widely evaluated using the 3C model [18], however, the model may not provide an insight into the structural or systemic issues affecting vaccination intention. A variety of factors are deemed to affect vaccine acceptance, and may be interacting at multiple levels [19]. Understanding social processes may be necessary to complement existing individual psychological models on vaccine hesitancy [20], and unearth key factors that could inform approaches to improve vaccination coverage. 

Following the COVID-19 vaccine rollout in Kenya, we set out to assess the acceptability of the COVID-19 vaccine among HCWs using a mixed methods approach. The study evaluated the predictors and factors driving vaccine acceptability using the WHO 3C model on quantitative data, and the 5C model on qualitative data. The findings may guide any improvements in vaccine deployment strategies in subsequent rounds of COVID-19 vaccinations, or in future vaccine-preventable epidemics.

## 2. Materials and Methods

### 2.1. Study Design

This is a concurrent mixed methods study, with cross-sectional data collected using an online survey, key informant interviews (KIIs) and focus group discussions (FGDs) with HCWs. This analysis is part of a larger multi-country study conducted in Ethiopia, Kenya and Nigeria, aimed at examining the COVID-19 vaccination policy and strategies, understanding HCWs’ vaccination disposition and capacity to promote the COVID-19 vaccination, and elucidate the population-level drivers of COVID-19 vaccine acceptance and hesitancy. This paper focuses on Kenya-specific data obtained from online surveys, FGDs and KIIs with HCWs.

### 2.2. Study Area and Population

The study’s target respondents were HCWs based at health facilities providing direct care to patients within the eight selected counties, namely, Nairobi, Kirinyaga, Kisumu, Machakos, Vihiga, Mombasa, West Pokot and Garissa. The counties were selected from a possible 47 counties to match the eight former provinces of Kenya and represent an estimated 23.6% of HCWs in Kenya. This approach ensures geographical representation across the country, and selection of health facilities in urban setup with large private and public ventures (Nairobi, Kisumu and Mombasa), peri-urban region with smaller public and private facilities (Kirinyaga, Machakos and Vihiga), and rural areas with marginalized regions and sparse public facilities (Garissa, Turkana). Respondents for the survey were obtained using a snowball approach, where the online survey link was shared randomly at health facilities across the eight counties and distributed among HCWs at that facility and through their professional networks. KII and FGD respondents were selected based on convenience sampling within target health facilities in the select counties, with a requirement that they be currently or recently (in the last one year) involved in the COVID-19 response, and provided direct patient services at health facility level. 

### 2.3. Instruments and Data Collection

Survey data were collected using a self-administered online questionnaire, focusing on participant demographics, vaccination behavior and intent, vaccine hesitancy, vaccination training and advocacy role. The questions were adapted from existing vaccine hesitancy and acceptability 3C model survey tool [21,22], with specific questions used highlighted in Table S1. The survey tool was programmed into Qualtrics software (Qualtrics XM, Provo, UT), and pilot tested in local non-study counties of Kiambu and Siaya in Kenya to assess clarity and applicability, with revisions made as required. The survey link was shared via WhatsApp messaging at random health facilities in the selected eight counties, and left active and monitored to reach the target sample size of at least 600 respondents. Survey data were collected between May and June 2022.

Qualitative KII guide collected data on attitude, knowledge and perception on COVID-19 vaccines, assessment of vaccination training, and role and drivers of vaccine advocacy, with the FGD guide designed to triangulate and validate the KII findings. The questions explored aspects of vaccine hesitancy/acceptability, and intended to validate findings from the survey. The KII and FGD guides were locally piloted using HCWs in non-study counties, with revisions made as required. Trained interviewers, employing both in-person and remote interviews via phone call or zoom conference call platform, conducted study interviews. All interviews were audio recorded and transcribed. The interviews were conducted between April and June 2022.

### 2.4. Data Analysis

For the quantitative component, participants who did not respond affirmatively to the gate consent question, or had incomplete survey record, or reported they were not HCWs, were excluded from the analysis. Descriptive analysis of the respondents was conducted based on demographic characteristics, vaccination behavior and intent. The respondents were categorized based on their responses to the 3C model questions. Exploratory factor analysis and correlation technique were employed to assess how well each of the items in the 3C survey questionnaire measured against the three constructs (confidence, complacency and convenience). Cronbach alpha was employed to ascertain the internal consistency reliability of the measurement items. An alpha value of 0.7 or higher indicated acceptable reliability. Items with poor loadings on each construct were dropped and the remaining items were indexed and summary score obtained. The association between the 3C model categorization, demographics and vaccination attitude as independent variables, and vaccination status as outcome variable, was determined using chi-square test. Simple and multiple predictor logistic regression analyses were performed with vaccination status as the outcome. To build the final model, associations between the variables were checked independently, and those that were significantly correlated were dropped. Variables that were determined to be significant (*p* < 0.05) in the univariate model were included in a multivariable analysis. Data analysis was conducted using R (R Core Team, 2020).

For the qualitative analysis, audio recordings were transcribed verbatim and non-English-based transcripts translated into English for analysis. Priori codes from similar interviews in Nigeria as part of the larger multi-country study employing the same interview guide were applied to develop the initial codebook. Further inductive coding was performed to generate insights unique to the Kenyan context. Different coders were used for the initial and subsequent coding. The findings were synthesized using the 5C model of vaccine hesitancy, also exploring contextual factors that were not a perfect fit to the model. Data analysis was conducted using NVivo (released in March 2020).

### 2.5. Ethical Considerations

The study was locally approved by Kenya Medical Research Institute, scientific and ethics review unit (SERU#4363), and by the Johns Hopkins Institutional Review Board (IRB00017765). Oral informed consent and electronic informed consent were obtained from all interview and survey respondents, respectively. Survey respondents who failed to respond or responded in the negative to the gate consent question were not allowed to take the online survey. Privacy and confidentiality were ensured through de-identification and secure storage of records.

## 3. Results

### 3.1. Baseline Characteristics

Of the 746 respondents included in this analysis, representation across the eight local counties was as follows: Kisumu (26%, n = 195), Nairobi (21%, n = 160), West Pokot (15%, n = 114), Garissa (11%, n = 79), Mombasa (9%, n = 67), Vihiga (8%, n = 56), Machakos (5%, n = 38) and Kirinyaga (5%, n = 37). The majority of the respondents were involved in patient counselling on COVID-19 vaccines (54%, n = 391), with a significant number also involved in vaccine administration (35%, n = 256), adverse event monitoring (20%, n = 150) and vaccine storage (19%, n = 140). The majority of the respondent HCWs were aged between 30 and 45 years (47%, n = 354), and working as clinicians and nurses (53%, n = 398) (Table 1). The respondents were mainly working in public health facilities (79%, n = 593), and within facilities offering COVID-19 vaccines (88%, n = 656). Based on 3C model classification, 78% of respondents were categorized as being confident about COVID-19 vaccines, 89% were non-complacent regarding the risk of acquiring COVID-19 and 88% perceived the vaccination service as being convenient for them.

### 3.2. Determinants of Vaccination Status

Table 1 also shows the distribution of respondents based on vaccination status, and the association with individual characteristics. Most respondents reported being partially or fully vaccinated against COVID-19 (92%, n = 685). Receipt of the vaccine was significantly associated with religion (χ^2^(1,n = 723) = 9.35, *p* = 0.041), being married (χ^2^(1,n = 724) = 10.13, *p* = 0.007), specific healthcare cadres (χ^2^(9,n = 746) = 57.58, *p* < 0.001), health facility type (χ^2^(2,n = 746) = 17.08, *p* < 0.001) and the availability of COVID-19 vaccines in their work facilities (χ^2^(1,n = 746) = 277.95, *p* < 0.001). Based on the 3C classification, vaccine receipt was more likely among those classified as confident about the vaccine (χ^2^(1,n = 746) = 94.62, *p* < 0.001), non-complacent regarding the risks of COVID-19 (χ^2^(1,n = 746) = 199.17, *p* < 0.001) and who found the vaccination services convenient (χ^2^(1,n = 746) = 252.33, *p* < 0.001).

### 3.3. Drivers of COVID-19 Vaccine Uptake

Of the HCWs partially or fully vaccinated, the majority reported that they were vaccinated to prevent them from catching COVID-19 (68%, n = 466) (Table 2). A smaller proportion reported they took the vaccine to protect their patients (11%, n = 78) or their family members (6%, n = 40). Generally, the respondents found it convenient to receive COVID-19 vaccines (96%, n = 660), and most reported they were extremely satisfied with the services received at the vaccination center (84%, n = 577).

### 3.4. Predictors of Vaccine Acceptability

In a multivariate model that included only the 3Cs, vaccination was more likely among those who were classified as finding the vaccine delivery service convenient (aOR 19.06, 95% CI 8.92–40.71, *p* < 0.001), non-complacent (aOR 9.42, 95% CI 4.38–20.27, *p* < 0.001) and confident (aOR 7.04, 95% CI 3.27–15.13, *p* < 0.001).

In terms of the addition of health facility availability of vaccines as an independent predictor to a model that contained only the 3Cs, there was a decrease in the strength of association for those classified as finding the vaccine delivery service convenient (aOR 5.35, 95% CI 2.08–13.74, *p* < 0.001). Independently, there was an association between health facilities providing vaccines and facility type (X^2^ (1,n = 746) = 42.95, *p* <0.001), and with those classified as finding the service convenient (X^2^ (1,n = 746) = 184.82, *p* < 0.001). Table 3 presents the final multivariable logistic regression model of the 3Cs, adjusting for the effect of the independent predictors of vaccine uptake. Vaccination was more likely to happen among those who found vaccination services to be convenient (aOR 20.13, 95% CI 9.01–44.96), who were not complacent about the risk of COVID-19 (aOR 10.15, 95% CI 4.64–22.22) and who were confident about the vaccine (aOR 6.37, 95% CI 2.90–14.02). Relationship status as an independent variable remained significant in the model, with vaccination more likely to happen among individuals in a marriage relationship (aOR 2.70, 95% CI 1.20–6.08). There was no significant association between those taking the vaccines and health facility type when adjusted for the effects of the 3Cs and relationship status.

### 3.5. Qualitative Findings

A summary of the characteristics of the KII respondents is detailed in Table 4 below. The three FGDs had five, six and eight HCWs as participants, the majority of whom were nurses.

### 3.6. Confidence

Vaccine safety: Most HCWs reported being motivated to take the vaccine because they felt confident that “it is safe” and “met the standards”. Those vaccinated reported that the vaccines did not hinder them from performing their daily functions, as the side effects experienced were mild and transient. The respondents also reported trust in the vaccines because they were being used in other jurisdictions and populations in the world. In addition, most respondents reported that education and training on the vaccines served to promote uptake. Knowledge of the vaccines and COVID-19 helped to demystify the infection. Training sessions were in the form of government initiatives, and from hospital-driven continuous medical education.

*“And then what made it even easier for us health care workers is because. A lot of CMEs like we call it continuous medical assessment where we, like we call people every weekend in the hospital, you tell them okay, now we’re giving Corona. It’s like, it’s like a seminar but within the hospital. Tell them now this week, we’re doing Pfizer. Next week we are changing to…, we kept on updating. So it made it easier for them to accept the vaccine”. (Source: KII with HCW, Mombasa)*

Vaccine efficacy: Most respondents expressed general confidence in the efficacy of the vaccines due to the observed reduction in the number of cases hospitalized and the corresponding mortality reported internationally following vaccination. Some noted that cases of infection were mild among vaccinated colleagues. However, for other respondents, COVID-19 vaccines’ efficacy presented an ambivalence of sorts. This was because, despite a belief in the vaccine’s protective effect, they witnessed COVID-19 infection cases in some of their vaccinated colleagues. Infections among vaccinated colleagues and community members eroded trust in the vaccine’s efficacy.

*“What made me think of being vaccinated and not being vaccinated is almost the same thing, because quite a number of vaccinated nurses and doctors caught COVID either admitted or not admitted, but they caught COVID… And if you get this, it’s where all of them were vaccinated. So actually, I was like, I get vaccinated, and I’m no longer sick. And I’m the same as those who are not vaccinated”. (Source: FGD with HCWs, Mombasa)*

Novelty: The fast pace with which the COVID-19 vaccines were developed and deployed compared to the time it took to develop other known vaccines contributed to doubts on the safety of the vaccines and raised further questions of rigor regarding the evaluation process. Relatedly, the approval of the COVID-19 vaccines for emergency use and prioritizing the frontline workers contributed to feelings that the vaccination was still experimental.

*“I have mixed emotions. Number one, you, you you’re not very, you know, the feeling of you’re the first ones on it. So are you privileged or are you a guinea pig? You know, that’s the balance, you have to decide in your mind and heart where you stand. So yeah, I think, for me, it was very interesting”. (Source: KII with HCW, West Pokot)*

Vaccine side effects: Whereas some respondents expressed fear of initiating vaccines due to the potential side effects, others reported that they were encouraged to take the subsequent doses because either they did not experience any side effects or because the side effects they experienced with the initial dose were already outlined and expected.

*“After being vaccinated, the first vaccine, I get the… I didn’t experience the side effects I used to hear from people say, and therefore I opted just to go for the second and finish the circle of vaccination”. (Source: FGD with HCWs, Mombasa)*

Trust in authorities: Some respondents expressed “trust in the profession” and confidence in the regulatory and approval authorities and agencies, indicating that once the approval was given, then it was definitely safe for human consumption. However, some respondents were skeptical regarding the intentions of the government and were less optimistic about the government’s ability to monitor safety.

*“Yeah, absolutely, you know, my trust in the profession, that those who are just as I am a surgeon, and my patients, trust me with their health care. So I, the same applies for my colleagues in virology and vaccine development… it was important that those who are there, give us the right information to make an informed choice”. (Source: KII with HCW, West Pokot)*

### 3.7. Convenience

Availability of vaccines: Most respondents reported that the availability of vaccines at most of the health facilities made it easier for them to access the vaccine. Respondents also reported higher coverage among staff in cases where the host institution was a vaccination center.

*“And then coming up with the various types of vaccine, it made it very easy for the people… they were some who wanted that for one day like JJ, some Pfizer etc.…In our institution. Three quarters of the staff have been vaccinated because the vaccination center was situated in our institutions. So they have been vaccinated”. (FGD with HCWs, Mombasa)*

Free of cost: Respondents also reported being motivated to take the vaccines because they were available free of charge and that the process of accessing the vaccines was transparent and straightforward, and devoid of any hindrances or complications for the HCWs.

*“Yeah, it’s there for us. I don’t know the exact I mean, I can’t quantify how many people go for that vaccination at the moment but it’s open and easily accessible”. (Source: KII with HCW, Kisumu)*

### 3.8. Complacency

Perceived susceptibility: Most HCWs felt that they were at risk of contracting COVID-19 infection due to close contact with patients; hence the need to protect themselves. This feeling was particularly heightened for providers who were working in COVID-19 isolation wards or caring for COVID-19 patients. For others, it was having family members or relatives who were at risk of being infected once they got it themselves.

*“Number one big factor here was the fact that I’m working in a high risk area both as in a hospital and more importantly as a surgeon. High risk aerosol and exposure to droplet infection. So, so that was a big motivator, of course. I’m a family man. So I have a responsibility to protect my family from my own workplace based infections. So that was also a big motivator”. (Source: KII with HCW, West Pokot)*

Perceived severity: Respondents also reported witnessing colleagues, friends and patients die from COVID-19-related complications; hence the drive to take the vaccine. Their knowledge of COVID-19 as a viral disease and having no known cure resulted in the vaccine being the only potential remedy available. The emergence of the COVID-19 variants of concern with varying degrees of virulence and transmissibility also motivated healthcare providers to take the vaccine and be able to protect themselves.

*“I worked in a COVID ward before I became a COVID nurse so I saw with all my two eyes, what used to happen to people. I saw how people used to struggle before they die. I saw so many people struggling for breath and for oxygen and fighting for their life. Really fighting. So it was very personal to me that nobody will tell me, nobody would tell me anything because I saw what happened. So whatever they’re telling me I know it’s something they heard or they think but me I know I saw one on one. So nothing would really discourage me from taking the vaccines actually nothing unless I just don’t want to but actually really nothing”. (Source: KII with HCW, Mombasa)*

### 3.9. Collective Responsibility

Some HCWs reported being motivated to take the vaccine because they were concerned about the potential risk they posed to their patients and wanted to protect their family members from workplace-acquired infections. For some, it was also about fulfilling the normative expectations of being a role model in the community for vaccine confidence. Safety for the rest of the population was also highlighted in their drive to attain herd immunity.

*“Secondly, be as an example to my environment, and also the people I’m interacting with, because maybe I’m serving a client and I’m encouraging the client to be vaccinated. So I have to act as a role model…also acting as a source of encouragement”. (Source: KII with HCW, Kisumu)*

### 3.10. Rational Calculation 

Some respondents reported being ambivalent about their intentions to vaccinate and did not possess strong attitudes relating to COVID-19 vaccines. Consequently, their vaccine uptake was influenced by reflections on the prevailing information regarding COVID-19 vaccines and drawing their own conclusions after assessing the potential risks versus benefits. For some HCWs, this quest for evidence-based decision making also meant observing vaccination outcomes of colleagues and relatives and making decisions based on whether they experienced severe outcomes or not.

*“I also depend a lot on evidence based decision making. So based on the research and the outcomes of, of the vaccine testing… I was not particularly concerned about the effectiveness. For me, it was a matter of what is more, what would be a worst consequence in terms of weighing the consequences of having it or not having it. And I think mine tilted to the balance of it being very costly not to have it”. (Source: KII with HCW, West Pokot)*

### 3.11. Contextual Factors

Vaccine mandates: The government’s requirement for all civil servants to be vaccinated was reported as a motivation by some HCWs to vaccinate in order to be compliant. In some instances, the motivation was about avoiding the inconvenience of being denied access to important services or places. Similarly, workplace pressure to conform and supervisors’ pressure to ensure compliance were reported as motivators for some. The restrictions on local and international travel were equally cited as a drive to vaccinate.

*“The bosses show us as an example, this was accepting the vaccine before even any other staff received it. … Then we were told, if we can’t have the vaccine, we will be fired to go back home… If you want the vaccine to retain your job or if you don’t want the vaccine, you are going back to the village”. (Source: KII with HCW, Mombasa)*

Rumors, misconceptions and conspiracy theories: Some of the rumors and conspiracy theories regarding COVID-19 infection and the vaccines influenced HCWs’ decision making and behavior regarding the vaccines. There were concerns that those who took the vaccine would soon die or that the vaccines would cause infertility. For some, COVID disease was a myth and this was a ploy by certain powerful players or stakeholders to make money given the country has other disease conditions of greater concern.

*“Big Pharma business, it is a business campaign, you know in Kenya COVID is still not the number one killer, still, we still have other diseases like malaria and HIV and TB, and cancer, those are the big boys in mortality rate in Kenya. COVID is still quite low. So, I mean, …why given so much attention, it’s because probably also it tends to affect people who are normally not being exposed by low socio economic conditions are getting COVID and those tend to be the wealthier. So, various reasons Big Pharma, government deals”. (Source: KII with HCW, West Pokot)*

Religious influence: Religious inclination influenced some HCWs’ attitudes towards COVID-19 vaccines. For some, this manifested in their belief that the COVID-19 pandemic and its existence is the will of God, and as such, whether one took the vaccine or not, they would still get infected. Relatedly, some churches took an official position regarding the COVID-19 vaccines’ safety and advocated against their adherents taking the vaccine.

*“I’m also a strong believer, Christian believer. So because of being a Christian, and I believe in God, I knew that this COVID has come and there is a reason or God has a purpose to allow it to be there. And if it is God’s plan, that you will get it you will get it whether you have been vaccinated or not. And if it is God’s plan that you don’t get the infection, whether you don’t vaccinate or you vaccinate, you will not get it. Being a believer, I also believed in that”. (Source: FGD with HCW, Mombasa)*

## 4. Discussion

High vaccine acceptance rates were observed among HCWs, with the majority being vaccinated to protect themselves and their family against COVID-19 infection. High vaccination intention rates for HCWs have been reported in China [23] and in South Africa [24], and were associated with doctor recommendation for patient vaccination and a higher level of education. This study was conducted one year after vaccine introduction, and expected vaccination intent to significantly improve after the COVID-19 vaccine’s distribution [25] as individuals evaluated the vaccine’s effect on others within the community before deciding to be vaccinated. A post-COVID-19 vaccine implementation survey among HCWs in Nigeria reported comparable high vaccination rates (92%), mainly attributed to vaccine confidence [26].

Applying the WHO 3C model in the analysis, the convenience of vaccination services was the biggest predictor of vaccine uptake among HCWs. Quantitative data show that convenience was mediated by the availability of vaccines at the specific health facility, and that most HCWs were satisfied with the services provided at vaccination centers. This is further supported by the qualitative data, where it was noted that working at vaccination centers might have resulted in a higher uptake among staff. Applying the 3C model to the general population in China, non-complacency and confidence had the greatest impact on COVID-19 vaccination intent, with non-complacency being the strongest determinant [27]. Other studies have shown a combination of confidence and collective responsibility as key determinants of COVID-19 vaccine acceptability to the public [25,28]. Among HCWs in Pakistan, moderate confidence and convenience were determined to be the strongest contributors to COVID-19 vaccine hesitancy [29]. In a review of evidence among the general population, the most common reason for not taking the COVID-19 vaccine was noted to be a lack of confidence in the vaccines, followed by complacency regarding the individual risk of being infected, and finally the lack of time to go and be vaccinated [30]. Possible reasons for the shift in significance of the three factors could be attributed to the difference in the level of knowledge when comparing HCWs to the public, and the fact that vaccine acceptability is largely contextual and changes over time [30,31]. Health-related habits of HCWs was determined to be different compared to the general population during the pandemic period [32]. Despite these differences, there is a possibility of other social and structural factors confounding these associations, hence, partly explaining the shifts. In this context, HCWs were largely targeted for COVID-19 vaccination training with several rounds of updates, and were prioritized for vaccine uptake with pressure from employers and colleagues alike.

Vaccine confidence is the highest predictor of vaccine uptake among physicians with regard to other adult-recommended vaccines [33,34], and even within routine childhood vaccination programs [35]. Despite significantly contributing to vaccine acceptance across many studies evaluating the intention to vaccinate, a population’s vaccine confidence has been shown to change over time and is easily influenced by safety scares and influential groups [36]. The study’s findings show that vaccine confidence was a significant factor in driving vaccine acceptance, although with a weaker strength of association compared to complacency and convenience. This could be related to changes in the constructs of vaccine confidence, which include trust in safety and effectiveness, the vaccine delivery system and the perceived intention of the policy makers [37]. An evaluation of COVID-19 vaccine hesitancy across 23 countries showed that HCWs were less concerned about vaccine efficacy [15], mainly attributed to knowledge. In a study among community health volunteers in Kenya [14], contextual influence including trust in the government delivery and risk management system was associated with COVID-19 vaccination intention. Most of the respondents were in government health facilities that also provided COVID-19 vaccines, and were more likely to be vaccinated. Association between healthcare system distrust and vaccine hesitancy has been documented and shown to be mediated by health literacy [38], which is likely to be worsened by the infodemic during the pandemic period. HCWs with their training and exposure to health information and the health system are more likely to assimilate the information and make an accurate determination on what is factual or not; hence, quickly building confidence in new vaccines as soon as they are rolled out. Increased knowledge on COVID-19 vaccines and the disease has been shown to be a positive predictor of vaccine acceptance by HCWs globally [11]. The qualitative data show that HCWs had some level of trust in vaccine regulatory approval and the authorities, and continued education and training resulted in the vaccine being perceived as safe and effective, with most side effects classified as expected based on prior knowledge. Notably, the biggest drive to confidence is observing first-hand the effects of the vaccine on infections and the adverse events related to it.

In a study evaluating influenza vaccine hesitancy among nursing home staff [39], complacency was most strongly associated with vaccination attitude, attributed to the misunderstanding of influenza’s severity and circulating strains. The quantitative data show that non-complacency is a significant factor in vaccine acceptability, with a stronger association than confidence. The qualitative data ascertained that HCWs working directly with COVID-19-infected patients at either isolation facilities or providing care led to risk realization. In addition, subsequent waves of infection resulting from variants of concern, and the impact of the same on colleagues and co-workers, heightened the drive to protect themselves and their family. These findings are similar to other studies that reported higher complacency among those who had recently cared for COVID-19 patients [40,41]. In addition, COVID-19 presented unique challenges to HCWs with threats of recurring infection and increased risk among specific cadres [6]. Those hesitant only about COVID-19 have been reported to differ from those who are normally hesitant about other vaccines [40].

The results show that being married was an individual predictor of vaccination uptake. Similar findings have been reported on COVID-19 vaccines in other parts of Africa [42]. Qualitative data show that HCWs were concerned about the risk of infecting their families; hence, they would vaccinate to reduce that risk. It is likely that pressure from family members or their spouse to be vaccinated influenced their individual decision. The promotion of social or familial norms with regard to vaccination has been shown to increase the uptake of childhood vaccination [43], indicating that social or professional support has a significant influence on vaccination behavior.

This study has several limitations. Firstly, being a cross-sectional study, we may not infer causality, as it is likely that other factors may have influenced vaccination. After being vaccinated, a perception on confidence, convenience and complacency may have developed. However, the study employed qualitative methods to try to elucidate some of these contextual factors, and discussed them using the broader 5C model. Relatedly, using existing models to focus analysis may leave out other significant contextual factors driving vaccination intent. Incorporating qualitative data more broadly might have identified some of the contextual and structural issues. Secondly, as much as the sample size was deemed adequate for a cross-sectional study, data collection was targeted to select HCWs from eight counties of the possible 47, who may not be truly representative of the entire HCW population across the country. However, it was noted that the survey was distributed through professional networks to HCWs beyond the target counties. It is also likely that this snowball approach may have biased the subsequently sampled respondents in certain ways, possibly along socio-economic groupings. Thirdly, the outcome of interest was self-reported vaccination status. Given existing mandates and pressure from HCW colleagues, it is likely that some HCWs may have provided socially desirable answers. This was likely limited as the survey link was distributed via phone, and the HCWs had the option to complete it in a private and safe space.

There is a need to understand the scale of COVID-19 vaccine acceptance among HCWs in specific contexts to develop tailored tactics to increase coverage. With the majority of HCWs initially being vaccinated, the reach for the “vaccine refusals” poses the greatest challenge, coupled with the need for booster doses across the whole population. Unvaccinated HCWs pose a risk of contracting COVID-19 infection or spreading it, negatively influencing the community and increasing vaccine hesitancy levels. This study determined that convenience, complacency and confidence are significant factors in promoting vaccine uptake, with convenience being most strongly associated. Further longitudinal studies are needed to determine the transition of these factors in the different phases of a pandemic, and how that may inform suitable interventions at the specific time point. The need to make rational decisions and protect family members were strong drivers for HCW vaccination. Other social factors including marital status, work place pressure, religion and misinformation have a role in influencing HCWs’ vaccination decisions. There is a need to periodically evaluate HCWs’ social environment in relation to vaccines when developing strategies to improve their acceptance and uptake of the vaccines.

## Figures and Tables

**Table 1:** Respondent’s sociodemographic and 3C classification according to vaccination status.

	Total (n = 746)	Non-Vaccinated (n = 61)	Vaccinated (n = 685)	*p*-Value ^a^
	N (%)	N (%)	N (%)	
**Religion ^b^**				
Muslim	76 (10%)	11 (14%)	65 (86%)	0.041
Christian	633 (85%)	44 (7%)	589 (93%)	
Traditional	13 (2%)	3 (23%)	10 (77%)	
Hindu	1 (0%)	0 (0%)	1 (100%)	
**Gender**				
Female	390 (52%)	31 (8%)	359 (92%)	0.812
Male	356 (48%)	30 (8%)	326 (92%)	
**Age**				
<30	324 (43%)	30 (9%)	294 (91%)	0.402
30–45	354 (47%)	28 (8%)	326 (92%)	
>46	68 (9%)	3 (4%)	65 (96%)	
**Current Marital Status ^c^**				
Married	402 (55%)	21 (5%)	381 (95%)	0.007
Unmarried	322 (45%)	34 (11%)	288 (89%)	
**Healthcare Cadre**				
Nurse	157 (21%)	13 (8%)	144 (92%)	<0.001
Community Health Worker	42 (6%)	2 (5%)	40 (95%)	
Public Health Officer	92 (12%)	5 (5%)	87 (95%)	
Clinician ^d^	241 (32%)	9 (4%)	232 (96%)	
Lab Technician	13 (2%)	0 (0%)	13 (100%)	
Health Records officer	32 (4%)	5 (16%)	27 (84%)	
Pharmacist	108 (14%)	27 (25%)	81 (75%)	
Counselor	14 (2%)	0 (0%)	14 (100%)	
Nutritionist	14 (2%)	0 (0%)	14 (100%)	
Other Specialties ^e^	33 (4%)	0 (0%)	33 (100%)	
**Health Facility Type**				
Non-Government	153 (21%)	25 (18%)	118 (83%)	<0.001
Government	593 (79%)	36 (6%)	557 (94%)	
**Facility Classification**				
Primary Level ^f^	431 (58%)	42 (10%)	389 (90%)	0.068
Secondary Level ^g^	231 (31%)	17 (7%)	214 (93%)	
Tertiary Levels ^h^	84 (11%)	2 (2%)	82 (98%)	
**Facility providing COVID-19 Vaccines**				
No	90 (12%)	48 (53%)	42 (47%)	<0.001
Yes	656 (88%)	13 (2%)	643 (98%)	
**Confidence**				
Not Confident	167 (22%)	44 (26%)	123 (74%)	<0.001
Confident	579 (78%)	17 (3%)	562 (97%)	
**Complacency**				
Complacent	83 (11%)	40 (48%)	43 (52%)	<0.001
Not Complacent	663 (89%)	21 (3%)	642 (97%)	
**Convenience**				
Not Convenient	86 (12%)	45 (52%)	41 (48%)	<0.001
Convenient	660 (88%)	16 (2%)	644 (98%)	

^a^ *p*-value for χ^2^ test. ^b^ Less than the total sample size as 23 respondents selected “Prefer not to say”. ^c^ Less than total sample size as 22 respondents selected “Prefer not to say”. ^d^ Includes medical doctors and clinical officers. ^e^ Includes physiotherapists, epidemiologists, radiographers, data analysts, clerks and medical students. ^f^ Includes health posts, dispensaries and community facilities. ^g^ Includes county general and specialist hospitals. ^h^ Includes teaching and referral hospitals or national/county referral hospitals.

**Table 2:** Reasons for COVID-19 vaccination among vaccinated healthcare workers (n = 685).

	**N (%)**
**Which of the following reasons reflects why you decided to get the COVID-19 vaccine?**	
To prevent me from getting COVID-19	466 (68%)
To prevent me from spreading the COVID-19 virus to my patients	78 (11%)
To prevent me from spreading the COVID-19 virus to my family	40 (6%)
Adding to the number of people in the community who are protected from getting COVID-19	30 (4%)
The vaccine was easily accessible and so I took it	26 (4%)
I live with someone/some people who belong to vulnerable populations	18 (3%)
My employer/government requires me to take the vaccine	17 (2%)
To get back to normal life	9 (1%)
Stop spreading the virus to others if I acquire it	1 (0%)
**How convenient was it for you to get the COVID-19 vaccines?**	
Extremely convenient	509 (74%)
Somewhat convenient	151 (22%)
Only a little convenient	20 (3%)
Not at all convenient	5 (1%)
**How satisfied were you with the service you received at the vaccination center?**	
Extremely	577 (84%)
Somewhat	99 (14%)
Only a little	8 (1%)
Not at all	1 (0%)

**Table 3:** Multivariate logistic regression model of predictors of vaccine acceptability.

	Unadjusted OR	*p*-Value	Adjusted OR	*p*-Value
Variable	(95% CI)		(95% CI)	
**Confidence**				
Not confident	Ref	<0.001	Ref	<0.001
Confident	11.83 (6.54–21.39)		6.37 (2.90–14.02)	
**Complacency**				
Complacent	Ref	<0.001	Ref	<0.001
Not Complacent	28.44 (15.43–52.43)		10.15 (4.64–22.22)	
**Convenience**				
Not Convenient	Ref	<0.001	Ref	<0.001
Convenient	44.18 (23.02–84.79)		20.13 (9.01–44.96)	
**Relationship status**				
Unmarried	Ref	0.002	Ref	0.016
Married	2.39 (1.38–4.14)		2.70 (1.20–6.08)	
**Health Facility Type**				
Non-public health facility	Ref		Ref	
Public health facility	3.02 (1.75–5.21)	<0.001	1.01 (0.42–2.40)	0.985

**Table 4:** Demographic characteristics of key informant interviewees.

	**Total (n = 18)**
	**N (%)**
**Age**	
<30	8 (44%)
30–45	9 (50%)
>46	1 (6%)
**Respondent Gender**	
Male	12 (67%)
Female	6 (33%)
**Respondent Role**	
Health worker at tertiary facility level	7 (39%)
Health worker at secondary facility level	7 (39%)
Health Worker at primary facility level	2 (11%)
Technical Officer (county)	2 (11%)
**Respondent Education**	
Graduate	16 (89%)
Post-graduate	2 (11%)
**Respondent Specialty**	
Nurse	6 (33%)
Doctor	2 (11%)
Clinical Officer	2 (11%)
Local Immunization Manager	1 (6%)
Pharmacist	1 (6%)
Other (not specified)	6 (33%)

## Data Availability

The data presented in this study are available on reasonable request from the principal investigator, C.W. The qualitative data are not publicly available due to the need to protect privacy and ethical concerns.

## Supplementary Materials

The following supporting information can be downloaded at: https://www.mdpi.com/article/10.3390/vaccines11081290/s1, Table S1: 3C Model Construct Questions and those Dropped by Exploratory Factor Analysis.
