# Supplementary material for: Determinants of COVID-19 Vaccine Acceptability among Healthcare Workers in Kenya—A Mixed Methods Analysis
Source: Vaccines (Basel). 2023 Jul 27;11(8):1290. doi: 10.3390/vaccines11081290 (PMC10459762; doi:10.3390/vaccines11081290)
Supplement: Supplementary file 1 [file vaccines-11-01290-s001.zip › vaccines-2435785-supplementary.pdf]

Table S1: 3C Model Construct Questions and those Dropped by Exploratory Factor Analysis.

**Constructs**	**Question Items**	**Remarks**
Confidence	I am completely confident that COVID-19 vaccines are safe.	
	I am confident that decisions regarding COVID-19 vaccination by public authorities (NPHCDA) is in the best interest of the community.	
	I am confident that decisions regarding COVID-19 vaccination by the Federal Government is in the best interest of the community.	
	COVID-19 vaccines are effective.	
	Medicines do more harm than good.	Dropped
Convenience	Everyday stress prevents me from being vaccinated.	
	The vaccination sites are too far from me.	
	The process of getting vaccinated is too stressful for me.	
	I don't have time off work to get vaccinated.	
Complacency	My immune system is so strong; it protects me against COVID-19 and I don't need to be vaccinated.	
	COVID-19 is not so severe that I should be vaccinated.	
	How likely is it that you are going to buy/use a recreational drug.	
	I think it is important to take warnings about negative outcomes seriously, even if the negative outcomes will not occur for many years.	Dropped
	I only act to satisfy immediate concerns, figuring that the future will take care of itself.	Dropped
	I only act to satisfy immediate concerns, figuring that the future will take care of itself.	Dropped
